# Editorial Notes

**Published:** 1945

**Authors:** 


### Editorial Notes

**The Society and The Journal.**

Now that the War is over the meetings of the Medico-Chirurgical Society will be resumed. Since the *Journal* exists primarily for publishing the Society's Transactions, the Editorial Committee looks forward to re-establishing the quarterly issue of this *Journal*. We owe a debt of gratitude to our readers and advertisers for their sympathetic forbearance of our shortcomings.

**Victory in Europe.**

ON Tuesday, May 8th, 1945, the Prime Minister, Mr. Winston Churchill, announced the surrender of Germany to the Allied Nations and the cessation of hostilities in Europe. No better account of this momentous occasion can be given than by quoting from the Prime Minister's statement to the House of Commons :—

" Yesterday morning at 2.41 a.m. at General Eisenhower's Headquarters, General Jodl, the representative of the German High Command, and of Grand Admiral Dönitz, the designated Head of the German State, signed the act of unconditional surrender of all German land, sea and air forces in Europe to the Allied Expeditionary Force and simultaneously to the Soviet High Command. . . . Our dear Channel Islanders will be free to-morrow. Hostilities will end officially at one minute after midnight to-night, Tuesday, the 8th of May, but in the interests of saving lives the ' cease fire ' began yesterday to be sounded on all fronts. . . . The German war is therefore at an end. After years of intense preparation, Germany hurled itself on Poland at the beginning of September, 1939, and in pursuance of our guarantee to Poland and in common action with the French Republic, Great Britain, the British Empire and Commonwealth of Nations declared war against this foul aggression. After gallant France had been struck down, we from this island and from our united Empire, maintained the struggle single-handed for a whole year until we were joined by the military might of Soviet Russia and later by the overwhelming power and resources of the United States of America. Finally, almost the whole world was combined against the evildoers who are now prostrate before us. Gratitude to our splendid Allies goes forth from all our hearts. We may allow ourselves a brief period of rejoicing, but let us not forget for a moment the toils and efforts that lie ahead. Japan, with all her treachery and greed, remains unsubdued. The injuries she has inflicted upon Great Britain, the United States and other countries and her detestable cruelties call for justice and retribution. We must now devote all our strength and resources to the completion of our tasks both at home and abroad. Long live the Cause of Freedom ! God Save the King ! "

**Regius Professor at Cambridge.**

H.M. THE KING has appointed Sir Lionel E. H. Whitby, C.V.O., to be Regius Professor of Physic in the University of Cambridge. This appointment has certainly one aspect of novelty, for the Chair of Physic has for very many years been occupied by clinicians. The brilliant work Sir Lionel Whitby has performed during the war, when he organized and commanded the Army Blood Transfusion Unit at Southmead, is thus fittingly recognized. The University of Cambridge may be congratulated on an appointment that will add further prestige to the Faculty of Medicine.

**Medical Director General R.N.** Surgeon Rear-Admiral H. St. C. Colson, C.B.E., until recently commanding officer at R.N. Auxiliary Hospital, Barrow Gurney, has been appointed Medical Director-General of the Royal Navy. In the war of 1914–18 this officer was decorated and promoted for his brilliant work during the Zeebrugge attack. His administrative abilities during the recent war have received the recognition they deserve. It will be a long time before our Society can again record at the same time such outstanding preferment for two of its members.

**Appointment of Professor of Surgery.** ROBERT MILNES WALKER, M.S., F.R.C.S., has been appointed Professor of Surgery in succession to Professor Rendle Short. Mr. Milnes Walker obtained Gold Medals in the M.B., B.S., and M.S. examinations in London University. After being Assistant in the Surgical Unit at University College Hospital, he was appointed Surgeon to the Royal Hospital, Wolverhampton. During the war he has acted as Group Adviser in Surgery in the E.M.S. Region 9.

**Hospital Survey of the South-West.** THE Survey of the Hospital Services of the South-Western Area (Region 7) has been published. It is a very complete report divided into two parts, like all the other Surveys. The first part contains full descriptions of hospital accommodation in the Counties of Gloucester, Somerset, Wiltshire, Devonshire and Cornwall, together with the County Boroughs of Bristol, Gloucester, Bath, Exeter and Plymouth, as it existed in Mid-1938. The total population at that date was estimated to be 2,645,980. The number of institutions dealt with was 298, distributed as follows :—

| | |
|---|---|
| Voluntary General Hospitals .. | 98 |
| Municipal General Hospitals .. | 4 |
| Voluntary Special Hospitals .. | 21 |
| Municipal Special Hospitals .. | 1 |
| Maternity Homes .. .. .. | 14 |
| Public Assistance Institutions .. | 48 |
| Isolation Hospitals .. .. .. | 34 |
| Tuberculosis Sanatoria, etc. .. .. | 20 |
| Convalescent Homes .. .. .. | 24 |
| Isolation Hospitals .. .. .. | 34 |

The various services participating in hospital work are all included in the Survey.

There are general observations on the geography and industries of the Region as well as on the structure, administration and general working of the hospitals ; the medical liaison between hospitals ; the pathological services ; the maternity services ; the tuberculosis services ; the isolation hospitals ; the institutions for chronic aged sick ; the convalescent homes ; and the ambulance services.

The second part of the Report contains the Surveyors' recommendations on the development of the hospital services. Readers of this Report must bear in mind that there are, in all, ten reports. covering the ten areas into which the whole of England and Wales is divided. To form a clear picture of the needs of the country the whole ten reports must be studied. It is a waste of time to regard a single report as foreshadowing the Government proposals for a new hospital service. The second part of this report sets forth what, in the opinion of the Surveyors, are the essential needs of the area and to this the Surveyors have added some of the ways of meeting these needs which, in their opinion, might be adopted. At this stage no criticism is called for. Everyone may now inform himself of the state of the hospital services as they exist at present. The facts ascertained by the Surveyors are not likely to be challenged or controverted. It is impossible that other investigators should enjoy similar facilities at the present time for conducting identical enquiries. The public, like the Minister of Health, must take the view that the facts are correctly stated. Moreover, since the Minister and his advisers will not formulate any plans until he and they have digested all the Surveys, the general public will be wise to wait for the proposals of the Minister before expressing any opinions. We must, at any rate, congratulate the Surveyors of the South-Western Area on having completed a magnificent task and having presented their report in a most readable form.

There is a striking conformity of views expressed in the various Surveys, more particularly in the planning of hospitals as satellites revolving about and depending on a main medical teaching centre. This pattern was not dictated by the Ministry of Health, nor by the Nuffield Trust. Evidently it is a pattern which has already begun to take shape and the Surveyors have recognised that it may well be accepted as a sound foundation on which to build.

**Selection of Medical Students.**

DR. BODMAN'S suggestions on the Selection of Medical Students are published at the request of the Bristol Study Group and the Committee of the Medico-Chirurgical Society. It is interesting to compare the views of to-day upon the catching, training and using of doctors with those of Dr. John Brown, the author of *Rab and His Friends*. He wrote (in 1882): "I am more convinced than ever of the futility and worse of the Licensing System, and think with Adam Smith, that a Mediciner should be as free to exercise his gifts as an Architect or Mole-catcher. The Public has its own shrewd way of knowing who should build its house or catch its moles, and it may quite safely be left to take the same line in choosing its doctor. Lawyers, of course, are different, as they have to do with the State—with the law of the land." Up to the present it has not been proposed to place Mole-catchers on a State Register.

